# Ultrasound-driven microbubble motors for targeted myocardial ischemia-reperfusion injury treatment

**DOI:** 10.1016/j.mtbio.2026.103349

**Published:** 2026-06-13

**Authors:** Mingyuan Xu, Jiaxin Shan, Haichao Yang, Jiaxu Wang, Haobo Yang, Jinhong Liu, Jianfeng Chen, Dandan Yu, Tiecheng Zhang, Meng Yang, Wei Zhang, Zhuo Wang, Xiaoping Leng

**Affiliations:** a Department of Ultrasound, the Second Affiliated Hospital of Harbin Medical University, Harbin, 150086, China; b Ultrasound Molecular Imaging Joint Laboratory of Heilongjiang Province (International Cooperation), Harbin, 150086, China; c Department of Ultrasound, the First Affiliated Hospital of Harbin Medical University, Harbin, 150001, China; d Key Laboratories of Myocardial Ischemia, Harbin Medical University, Ministry of Education, Harbin, 150086, China; e Laboratory Animal Center, the Second Affiliated Hospital of Harbin Medical University, Harbin, 150086, China; f Department of Magnetic Resonance Diagnosis, the Second Affiliated Hospital of Harbin Medical University, Harbin, 150086, China; g Department of Ultrasound, Beijing Tiantan Hospital, Capital Medical University, Beijing, 100070, China; h State Key Laboratory of Frigid Zone Cardiovascular Diseases (SKLFZCD), Harbin Medical University, Harbin, 150086, China

**Keywords:** Neutrophil membrane biomimicry, Ultrasound-driven, Mobile micro-/nano motors, Myocardial ischemia-reperfusion injury, Cyclosporine A

## Abstract

Myocardial ischemia-reperfusion injury (MIRI) is a secondary pathological process that occurs after restoration of blood flow during the treatment of acute myocardial infarction. The effective prevention of MIRI is pivotal for optimizing therapeutic efficacy and improving long-term patient outcomes. In this study, we developed an ultrasound-driven, multilevel targeted bionic microbubble motor (CsA@NM-MBs motors) that can break through the vascular endothelial and cell membrane barriers to achieve precise, targeted drug delivery. The CsA@NM-MBs motors were coated with a neutrophil membrane, a highly biocompatible membrane with natural inflammatory targeting abilities, enabling active homing to damaged areas. Upon application of an external ultrasound field, the CsA@NM-MBs motors actively crossed the vascular endothelial barrier and reached the myocardial tissue in the MIRI area with precision. With the aid of ultrasound-targeted microbubble destruction (UTMD) technology, acoustic pores are formed on the cell membrane, enabling it to break through the cell barrier. The driving force generated by the explosion enables CsA to be delivered into cells, thereby achieving safe and efficient drug release. This ultrasound-driven cell membrane biomimetic multilevel targeting strategy significantly enhanced the enrichment and delivery efficiency of CsA at the injury site, providing a new approach for the treatment of MIRI.

## Graphical abstract

Scheme 1. Schematic illustration. The schematic illustration provides a detailed depiction of the structural composition and targeting mechanism of CsA@NM-MBs motors, as well as their therapeutic efficacy in the treatment of MIRI. The underlying process involves three key steps: ①neutrophil membranes enable targeted delivery of CsA@NM-MBs to inflamed endothelial cells at the MIRI site; ②ultrasound-driven propulsion facilitates transendothelial transport, allowing CsA@NM-MBs motors to penetrate the endothelial barrier and reach the injured myocardial tissue; ③UTMD induces localized disruption of CsA@NM-MBs motors, leading to the controlled release of CsA for effective therapeutic intervention.

**Figure:**
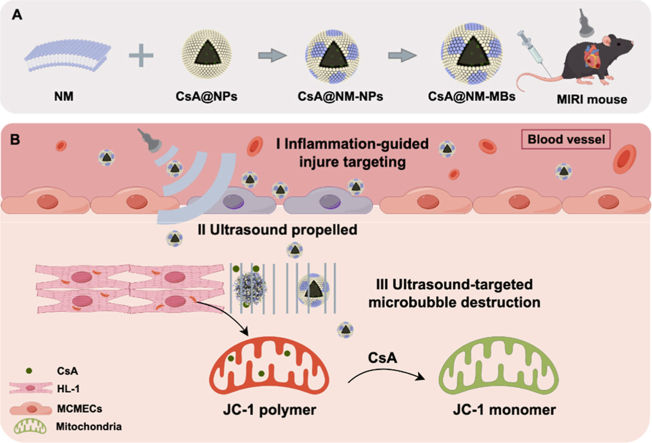

## 1 Introduction

Acute myocardial infarction remains the leading cause of global mortality [1]. Reperfusion therapy, the cornerstone of clinical management, effectively restores blood flow to the ischemic myocardium and limits infarct progression [2,3]. However, the reintroduction of circulation paradoxically triggers myocardial ischemia/reperfusion injury (MIRI), characterised by exacerbated cellular apoptosis and necrosis, which undermines the therapeutic benefits of reperfusion and adversely affects long-term patient outcomes [4,5]. Consequently, the development of effective interventions to mitigate MIRI is critical for enhancing the overall success of acute myocardial infarction treatment. Cyclosporine A (CsA) has emerged as a promising therapeutic agent for MIRI, with early clinical evidence demonstrating its ability to reduce infarct size by approximately 20% in patients with acute myocardial infarction patients through the selective inhibition of mitochondrial permeability transition pore opening, a key pathway in cell death^[^[6], [7], [8]^]^. Despite this potential, the clinical utility of CsA is limited by its poor aqueous solubility and low bioavailability, necessitating high systemic doses that increase the risk of immunosuppression and off-target cytotoxicity [9,10]. Thus, achieving targeted accumulation of CsA specifically within the reperfused injury zone with localised potency and low systemic toxicity, represents a pivotal challenge that must be overcome to fully realise its therapeutic potential in MIRI.

Micro/nanomotors can convert external energy into autonomous mechanical motion [11]. Unlike conventional passive diffusion-based carriers, these motors enable directional and controllable locomotion in complex biological environments through actuation by external energy fields such as magnetic, acoustic, or chemical stimuli [12,13]. Ultrasound-driven propulsion is a particularly promising approach owing to its fuel-free operation, deep tissue penetration, and remote on-demand activation. This mechanism relies on acoustic radiation forces and acoustic streaming effects to modulate the surrounding fluid dynamics, thereby enabling the rapid and precise navigation of micro-nanomotors [14,15]. Among these, ultrasonically driven biomimetic microbubble motors have emerged as highly effective platforms for targeted drug delivery. By cloaking the motor surface with cell membranes, a biomimetic system endowed with dual functionalities—immune evasion and lesion-specific targeting—can be engineered. Under an acoustic radiation force, these motors directionally migrate along the ultrasound propagation axis, efficiently traversing vascular endothelial barriers. Upon reaching the target site, localised ultrasound stimulation triggers cavitation effects that not only facilitate spatiotemporally controlled drug release but also transiently disrupt cellular membranes, enhancing intracellular delivery. By transforming passive diffusion into active multilevel targeting, ultrasound-driven biomimetic microbubble motors significantly enhanced myocardial drug enrichment while minimising systemic exposure, offering a novel technological pathway toward precision medicine [16].

In this study, we developed an ultrasound-driven microbubble motor for targeted drug delivery to injured myocardial tissues for the treatment of MIRI. The fabrication strategy involved encapsulating CsA, a therapeutic agent with established cardioprotective effects in liposomal nanoparticles (NPs), followed by coating the surface with neutrophil membranes via sonication. This biomimetic modification enabled the system to harness the innate inflammatory chemotaxis of neutrophils [17,18]. The phase-change agent perfluoropentane (PFP), encapsulated within liposomes, undergoes a liquid-to-gas transition upon exposure to low-intensity focused ultrasound (LIFU), thereby generating microbubbles [19]. These microbubbles facilitate both ultrasound-guided propulsion. The resulting neutrophil membrane-camouflaged ultrasound-responsive system was designated as the CsA@NM-MBs motors. The neutrophil membrane coating enhances biocompatibility, allowing the motors to evade immune clearance without eliciting significant immunogenic responses [20] while preserving the intrinsic ability of neutrophils to target inflamed tissues [21], thereby achieving precise accumulation at the site of myocardial injury. Upon reaching the lesion, continuous ultrasound actuation generates acoustic radiation forces that drive the directional movement of microbubbles, facilitating transendothelial transport across the vascular barrier and enabling deep penetration into the myocardium to core of the injury. Concurrently, ultrasound-triggered cavitation induces transient and reversible pore formation in cell membranes and endothelial barriers, enhancing paracellular and transcellular permeability. During this process, the microbubble shell fragments and degrades, ensuring the rapid and efficient release of CsA, which subsequently exerts its cardioprotective effects by inhibiting the opening of the mitochondrial permeability transition pore. This innovative neutrophil-mimicking, ultrasound-driven microbubble motor system integrates multilevel targeting, active transport, and stimuli-responsive drug release, offering a promising therapeutic strategy for MIRI with high clinical translation potential.

## 2 Materials and methods

**Isolation of neutrophils:** All animal experiments were performed in strict accordance with the guidelines approved by the Ethics Committee of the Second Affiliated Hospital of Harbin Medical University. Neutrophils were isolated from the bone marrow of male C57BL/6 mice using density gradient centrifugation. The mice were euthanized and immersed in 75% ethanol for 5 min to disinfect the surface. The femurs and tibias were then aseptically dissected, and the epiphyseal ends removed to expose the marrow cavity. The bone marrow was flushed out with Roswell Park Memorial Institute 1640 medium (Cat. No. 11875500, Gibco, Thermo Fisher Scientific, Carlsbad, CA, USA) using a sterile syringe. The resulting cell suspension was filtered through a 70 μm cell strainer to remove tissue debris and clumps. A commercial neutrophil separation solution (P8370, Solarbio, Beijing, China) was diluted with physiological saline to prepare 71% and 61% (v/v) solutions. In a sterile 15 mL centrifuge tube, 3 mL of the 71% solution was carefully layered underlaid with 3 mL of the 61% solution to establish a discontinuous density gradient. The filtered bone marrow suspension was gently layered on top of the gradient and centrifuged at 3000 rpm for 30 min with the brake disengaged. Following centrifugation, the neutrophil-enriched band located at the interface between the two layers was carefully aspirated using a sterile pipette. The erythrocytes were lysed by resuspending the cell pellet in 3 ml of red blood cell lysis buffer for 5 min, followed by centrifugation at 500 × g for 5 min. The resulting pellet was washed once with phosphate-buffered saline (PBS) and resuspended in the appropriate culture medium for subsequent experiments.

**Extraction of neutrophil membranes:** Neutrophil plasma membranes were isolated using the Mem-PER™ Plus Membrane Protein Extraction Kit (Cat. No. 89842, Thermo Fisher Scientific, Waltham, MA, USA) according to the manufacturer's instructions. Briefly, neutrophils were pelleted by centrifugation at 300 × g for 5 min at 4 °C. The supernatant was discarded, and the cell pellet was resuspended in 3 mL of wash buffer by gentle pipetting. The suspension was then centrifuged again under identical conditions (300 × g, 5 min). Following removal of the supernatant, the pellet was resuspended in 1.5 mL of wash buffer, transferred to a 2 mL microcentrifuge tube, and centrifuged once more (300 × g, 5 min, 4 °C). After the final wash, the cell pellet was resuspended in 0.75 mL of permeabilization buffer (provided in the kit), incubated on ice for 10 min with gentle agitation, and subsequently centrifuged at 16,000 × g for 15 min at 4 °C. The supernatant—containing soluble cytoplasmic proteins—was carefully removed, and the resulting membrane-enriched pellet was collected and stored at −80 °C until further use.

**Characterization of neutrophils:** Neutrophil morphology was assessed using Wright-Giemsa staining. Briefly, isolated neutrophils were resuspended in PBS, and a small aliquot was evenly smeared onto a glass slide. The smear was air-dried completely at room temperature prior to staining. Wright-Giemsa staining solution (Cat. No. G1020, Solarbio, Beijing, China) was applied to fully cover the smear and incubated for 1–2 min. Sodium phosphate buffer (pH-adjusted to 6.8–7.2) was added to dilute the stain and facilitate uniform color development, followed by an additional incubation of 3–5 min. The slide was then gently rinsed with distilled water to remove excess dye and air-dried before microscopic examination using a bright-field optical microscope. Neutrophil purity was evaluated by flow cytometry. Freshly isolated mouse neutrophils were resuspended in PBS containing 2% fetal bovine serum (FBS) to minimize non-specific antibody binding. Cells were incubated with fluorescein isothiocyanate (FITC)-conjugated anti-CD11b antibody (Cat. No. 101205, BioLegend, 1:100 dilution) and allophycocyanin (APC)-conjugated anti-Ly6G antibody (Cat. No.127613, BioLegend, 1:100 dilution) for 30 min at 4 °C in the dark. After incubation, cells were washed twice with ice-cold PBS to remove unbound antibodies. Fluorescence signals were acquired on a flow cytometer, and data were analyzed using FlowJo V10 software to determine the percentage of CD11b^+^Ly6G^+^ double-positive cells, which represent mature neutrophils.

**Preparation of CsA@NPs:** NPs and CsA@NPs were prepared using a combined thin-film hydration and ultrasonic emulsification technique [22]. Briefly, DSPC (10 mg; Avanti Polar Lipids), DSPE-PEG (4 mg; Avanti Polar Lipids), PG (3 mg; Avanti Polar Lipids), and CsA (2.5 mg; MedChemExpress) were co-dissolved in 10 mL of dichloromethane. The organic solvent was removed under reduced pressure using a rotary evaporator to yield a uniform, semi-transparent lipid film. The film was then hydrated with 10 mL of deionized water at room temperature, followed by bath sonication to ensure complete dispersion and formation of a homogeneous lipid suspension. To generate nanoemulsions, 600 μL of PFP was added dropwise under continuous ice-bath cooling. The mixture was subjected to probe sonication (60 W, pulsed mode: 5 s on/5 s off) for 2 min. The resulting emulsion was centrifuged at 12,000 rpm for 15 min at 4 °C using a refrigerated centrifuge. The pellet was collected and resuspended in deionized water to remove CsA and other unencapsulated components.

**Preparation of CsA@NM-MBs:** To prepare CsA@NM-NPs, the protein concentration of isolated neutrophil membranes was quantified using a BCA protein assay kit. Neutrophil membranes were then fused onto CsA@NPs at a membrane protein-to-phospholipid mass ratio of 2:1. Membrane fusion was facilitated by probe sonication under ice-cooled conditions. Following sonication, the suspension was centrifuged at 12,000 rpm for 15 min at 4 °C; the resulting pellet was washed and resuspended in deionized water to remove unincorporated, unfused membrane components. Finally, the CsA@NM-NPs suspension was subjected to trigger a low-intensity focused ultrasound irradiation to induce phase transition into CsA@NM-MBs.

**Characterization of CsA@NM-MBs:** The particle size, polydispersity index (PDI), zeta potential, and colloidal stability of NPs, CsA@NPs, CsA@NM-NPs and CsA@NM-MBs were measured using a Malvern laser particle sizer, with each sample analyzed in triplicate. Morphological analysis was conducted by transmission electron microscopy (TEM). Briefly, particles suspensions were appropriately diluted and deposited onto carbon-coated copper grids, followed by incubation at room temperature for 20 min. Excess liquid was carefully blotted with filter paper, and the grids were stained with 2% phosphotungstic acid for 5 min. After rinsing thoroughly with deionized water to remove residual stain, the grids were dried overnight under vacuum in a desiccator. Samples were subsequently imaged by TEM to evaluate morphology, size distribution, and structural integrity.

**Characterization of the neutrophil membrane:** Assessment of fusion efficiency between neutrophil membranes and liposomal membranes. To evaluate membrane fusion, neutrophil membranes were labeled with the lipophilic red fluorescent dye DiI, whereas liposomes were pre-labeled with the green fluorescent dye DiO. Neutrophil membranes were incorporated into CsA@NPs at a protein-to-phospholipid mass ratio of 2:1 and fused via probe sonication under ice-cooled conditions to generate CsA@NM-NPs. Fluorescence co-localization was quantified using fluorescence microscope and flow cytometry to determine the extent of membrane integration. Verification of neutrophil membrane protein retention and functional integrity. The presence of neutrophil membrane-associated proteins was confirmed by Coomassie Brilliant Blue staining and Western blotting. Protein samples were mixed with SDS-PAGE loading buffer (1:4, v/v) and denatured at 100 °C for 10 min in a heating block. Proteins were resolved by electrophoretically on precast SDS-PAGE gels and visualized by Coomassie Brilliant Blue staining following a 30 min incubation, followed by destaining until distinct bands were clearly resolved. For Western blotting, proteins were electrophoretically transferred from the gel to a PVDF membrane using either a semi-dry or wet transfer system. The membrane was blocked with a protein-free rapid blocking buffer for 1 h at room temperature, washed three times with TBST, and then incubated overnight at 4 °C with primary antibodies against key neutrophil surface markers: CD11b (AF7018, Abcam), CD29 (ab179472, Abcam), LFA-1 (DF6896, Affinity Biosciences), CXCR2 (DF7095, Affinity Biosciences) and GAPDH (AF7021, Affinity Biosciences). After three TBST washes, the membrane was incubated with horseradish peroxidase-conjugated goat anti-rabbit IgG secondary antibody (S0001, Affinity Biosciences) for 1 h at room temperature. Following three additional TBST washes, immunoreactive bands were detected using enhanced chemiluminescence reagent and visualized with a gel documentation system.

**Drug loading capacity:** CsA was prepared as a series of standard solutions at concentrations ranging from 0.01 to 0.1 mg/mL. Absorbance values were measured at 210 nm using a UV-visible spectrophotometer. A calibration curve was constructed by plotting absorbance (arbitrary units) versus CsA concentration (mg/mL), and the corresponding linear regression equation was used to quantify CsA content. For sample analysis, CsA@NM-MBs were centrifuged to separate the supernatant, which contained unencapsulated (free) CsA. The concentration of free CsA in the supernatant was determined using the established calibration curve. Encapsulation efficiency (EE) and loading efficiency (LE) were calculated using the following equations:

LE (%) = [(Total drug amount – Free drug amount) / Total particle mass] × 100%

EE (%) = [(Total drug amount – Free drug amount) / Total drug amount] × 100%

**Detection of phase-transition capability of CsA@NM-NPs:** An in vitro imaging model was established using a 2% agarose gel solution. A suspension of CsA@NM-NPs was loaded into designated wells, with PBS was added to control wells. LIFU irradiation was applied in pulsed mode with a focal length of 1.5 cm, a duty cycle of 50%, and an acoustic intensities of 2 W/cm^2^. Irradiation durations were set at 2, 4, 6, and 8 min. B-mode ultrasound and CEUS images were acquired in real time. The grayscale intensity within a defined region of interest in each image was quantitatively analyzed using ImageJ software to assess echogenicity changes associated with the phase transition.

**Motion of CsA@NM-MBs motors:** The ultrasonic driven system consists of a signal generator, a power amplifier, an ultrasonic transducer, and a sample chamber. The signal generator produces electrical signals, which are amplified by the power amplifier. The amplified electrical signals are then converted into mechanical ultrasound waves by the transducer, thereby actuating the CsA@NM-MBs motors within the sample chamber. Real-time observation of micromotor motion is performed using an inverted optical microscope (Olympus BX31), and videos are recorded with NIS-Elements AR 4.3 software. Subsequent trajectory analysis is conducted using the TrackMate plugin in ImageJ to track individual particle trajectories, extract frame-by-frame positional coordinate (x, y), and quantify key motility parameters-including instantaneous velocity, mean square displacement (MSD), and effective diffusion coefficient.

The MSD is calculated according to the following equation:

MSD = Average{(Xn-X1×Pixel)^2^ + (Yn-Y1×Pixel)^2^}

The average velocity (v) is determined using the formula:

$$v=\frac{\sqrt{\left(\text{Xn}‐X1\right)\times {\text{Pixe}l}^{2}+\left(\text{Yn}‐Y1\right)\times {\text{Pixe}l}^{2}}}{\Delta t}$$

To achieve precise on-off control of motor motion, the ultrasonic field is alternately activated (5 s) and deactivated (5 s) via the driving system. To investigate frequency-dependent motility, the driving frequency is systematically varied from 50 kHz to 100 kHz in 10 kHz increments. For each frequency, motor trajectories are recorded over a 5 s interval, and the corresponding average speed is quantified from the trajectory data. CsA@NM-MBs motors are individually dispersed in three physiologically relevant media: PBS, fetal bovine serum (FBS), and whole blood. These media were selected to evaluate motors performance under biologically diverse conditions-specifically, variations in viscosity, protein concentration, and cellular components-thereby enabling a rigorous assessment of the robustness and controllability of ultrasound-driven propulsion across complex biological matrices.

**Transmigration capacity across an inflamed endothelial barrier:** A Transwell assay was established to evaluate the ability of motors to cross the endothelial barrier. Mouse cardiac microvascular endothelial cells (MCMECs) were seeded onto 0.4 μm polycarbonate membrane inserts and cultured until confluent monolayers formed, serving as an in vitro model of the vascular endothelium. To mimic cardiomyocyte uptake of the motors, HL-1 cardiomyocytes were seeded in the lower chamber. Upon achieving 100% confluence, the chemoattractant N-formylmethionyl-leucyl-phenylalanine (fMLP) was added to the lower chamber to simulate an inflammatory microenvironment in vivo, thereby enabling assessing of the motors’ inflammatory targeting capability. DiO-labeled motors were then introduced into the upper chamber. At predetermined time points, fluorescence intensity in the lower chamber was quantified to determine the extent of motors transmigration across the endothelial monolayer.

**In vitro safety assessment:** To evaluate the biocompatibility of motors for potential in vivo applications, hemolytic activity was assessed using whole blood collected from the inferior vena cava of healthy mice. Blood samples were drawn using a sterile disposable needle and immediately transferred into EDTA-K2 anticoagulant tubes to prevent coagulation. Dilute whole blood with physiological saline at a 1:50 vol ratio to prepare a 2% (v/v) red blood cell (RBC) suspension. Aliquots (1 mL) of the RBC suspension were distributed into microcentrifuge tubes and assigned to the following groups: negative control (PBS), positive control (distilled water), NPs, CsA@NPs, CsA@NM-NPs, and CsA@NM-MBs. To each tube, 0.5 mL of PBS (negative control), distilled water (positive control), or particle dispersion (experimental groups) was added. The mixtures were gently vortexed and incubated at 37 °C for 3 h under gentle agitation to ensure uniform interaction. After incubation, samples were centrifuged at 2000 rpm for 5 min, and the supernatants were collected. Hemoglobin release was quantified by measuring absorbance at 545 nm using a UV-Vis spectrophotometer. Each sample was measured in triplicate, and the hemolytic index (HI) was calculated as follows:

HI = (A − B) / (C − B) × 100%,

where A denotes the absorbance of the test group, B represents the absorbance of the negative control (indicating spontaneous hemolysis), and C corresponds to the absorbance of the positive control (indicating complete lysis).

For cytotoxicity evaluation, H9C2 rat cardiomyoblasts in the logarithmic growth phase were harvested and suspended in Dulbecco's Modified Eagle Medium (DMEM) at a density of 1 × 10^5^ cells/mL. A 100-μL aliquot of the cell suspension was seeded into each well of a 96-well plate and incubated for 24 h under standard conditions (37 °C, 5% CO_2_) to allow full cell attachment. Subsequently, the culture medium was carefully aspirated, and the monolayer was washed twice with sterile PBS. Fresh DMEM complete medium was then added to all wells. Particle formulations—including NPs, CsA@NPs, and CsA@NM-NPs, CsA@NM-MBs-were added to designated wells at a concentration of 1 × 10^5^ particles per well and co-incubated with cells for an additional 24 h. Untreated control wells received no particles, while blank control wells contained only 100 μL of complete medium-without cells or test agents. Following treatment, the medium was aspirated, and cells were washed three times with PBS. Cell viability was determined using a commercially available assay kit according to the manufacturer's instructions. The relative cell survival rate was calculated with relative to the untreated control group to quantitatively evaluate the cytotoxic effects of each formulation.

**In vitro macrophage evasion capability:** To assess the ability of motors to evade phagocytic uptake, NPs, CsA@NPs, and CsA@NM-MBs were labeled with the lipophilic fluorescent dye DiO. RAW 264.7 macrophages were seeded into 24-well plates at an appropriate density and allowed to adhere prior to experimentation. Subsequently, 200 μL of each particle suspension was added to individual wells and gently mixed by orbital shaking to ensure uniform dispersion in the culture medium. After 1 h of incubation at 37 °C under 5% CO_2_, the medium was aspirated, and cells were fixed with 4% paraformaldehyde. Subsequently, cells were permeabilized with 1% Triton X-100 for 30 min at room temperature, followed by staining of the action cytoskeleton with phalloidin conjugated to a fluorophore (e.g., Alexa Fluor-conjugated) and nuclear counterstaining with DAPI. Particles localization relative to actin filaments and nuclei was visualized using fluorescence microscopy.

**Mitochondrial membrane potential assessment:** H9C2 cells were seeded in 6-well plates at a density of 5 × 10^4^ cells/mL (2 mL per well). After confirming cell attachment, the culture medium was aspirated and replaced with 2 mL of hypoxia-inducing medium per well. The plates were then incubated in a hypoxic chamber (95% N_2_ and 5% CO_2_) for 3 h. Subsequently, the medium was removed, and the cells were treated with fresh serum-free medium containing either CsA or CsA@NM-MBs under normoxic conditions for 4 h. Thereafter, the medium was aspirated, and the cells were washed three times with 1 × JC-1 Assay Buffer. For mitochondrial membrane potential measurement, cells were incubated with JC-1 working solution at 37 °C for 20 min in a humidified incubator. Following incubation, the JC-1 solution was carefully removed, and the cells were washed an additional three times with 1× JC-1 Assay Buffer prior to fluorescence microscope imaging.

**Mouse model of MIRI:** Male C57BL/6 mice (6–8 weeks old) were anesthetized with 2% isoflurane in oxygen. A left thoracotomy was performed via a 2-mm vertical incision made 2–3 mm lateral to the left sternal border. The intercostal muscles were carefully dissected using fine forceps to expose the pleura, which-along with the pericardium-was gently opened under direct visualization. The left anterior descending coronary artery (LAD) was ligated 3 mm distal to its origin using a 6/0 silk suture. Successful occlusion was confirmed by immediate and visible blanching of the anterior left ventricular wall. The heart was then gently repositioned into the thoracic cavity, intrathoracic air was evacuated, and the chest wall was closed with 4-0 absorbable sutures. After 30 min of ischemia, the ligature was released to initiate reperfusion [23]. Five min prior reperfusion, mice received a tail-vein injection of PBS, CsA, CsA@NM-NPs, or CsA@NM-MBs. Sham-operated control mice underwent identical surgical procedures except for LAD ligation and received an equivalent volume of saline. All animal experimental procedures were conducted in strict accordance with international ethical guidelines. This study was approved by the Animal Management and Use Ethics Committee of the Second Affiliated Hospital of Harbin Medical University (Ethics Number: YJSDW2023-235).

**Determination of CsA Using HPLC-MS/MS:** CsA was extracted from whole blood and tissue samples via protein precipitation and quantitatively analyzed by high-performance liquid chromatography–tandem mass spectrometry (HPLC-MS/MS). Chromatographic separation and mass spectrometric detection were performed on an AB Sciex API 4000™ LC-MS/MS system. Chromatographic separation was achieved on an ACE Excel 5C18 column (50 × 2.1 mm, 5 μm) using a mobile phase composed of solvent A (water containing 10 mmol/L ammonium acetate) and solvent B (acetonitrile containing 0.1% formic acid), delivered at a flow rate of 0.4 mL/min. Gradient elution was conducted as follows: 0 min, A:B = 50:50 (v/v); 0.1–3.0 min, A:B = 0:100; 3.1–5.0 min, A:B = 50:50. The injection volume was 10 μL; the column temperature was maintained at 40 °C; and the autosampler tray temperature was set to 4 °C. Mass spectrometric detection was carried out using an electrospray ionization (ESI) source operating in positive ion mode. High-purity nitrogen was used as both the sheath gas (35 arbitrary units) and the auxiliary gas (15 arbitrary units), while high-purity argon served as the collision gas (1.5 mTorr). Key MS parameters were optimized as follows: spray voltage, 5.5 kV; capillary temperature, 300 °C; selected reaction monitoring (SRM) scan width, 0.5 *m*/*z*; dwell time per transition, 0.2 s; and Q1 and Q3 peak widths, 0.7 *m*/*z*. A stock solution of CsA was prepared by dissolving the analyte in methanol to yield a concentration of 1 mg/mL. Working standard solutions at concentrations of 3200, 1600, 800, 400, 200, 100, and 50 ng/mL were generated by serial dilution of the stock solution with methanol. Cyclosporin D (CsD) was employed as the internal standard. Acetonitrile was used as the protein-precipitating agent. The calibration curve was constructed by plotting the peak area ratio (CsA/CsD) against nominal CsA concentration. Linear regression yielded the equation y = 0.003762x + 0.0573 (R^2^ = 0.9961). Drug concentration data were analyzed using a non-compartmental pharmacokinetic model and processed with DAS 2.0 software (Drug and Statistics for Windows, Version 2.0).

**In vivo targeting evaluation:** To assess the in vivo targeting efficacy of the particles, equal volumes of PBS, DiR-labeled CsA@NM-NPs, or DiR-labeled CsA@NM-MBs were administered intravenously via the tail vein to mice with MIRI. Fluorescence images of the excised hearts were acquired at 30 min, 2 h, and 24 h post-injection using an IVIS Spectrum imaging system (Caliper Lifesciences, USA).

**Cardiac function assessment:** Transthoracic echocardiography was performed using a Samsung RS80A ultrasound system equipped with an L7-16 probe to evaluate cardiac function in mice subjected to MIRI. Prior to imaging, mice were anesthetized with an isoflurane-oxygen mixture. Two-dimensional short-axis views of the left ventricle were acquired, followed by M-mode recordings at the level of the papillary muscles. Left ventricular function parameters-including left ventricular ejection fraction (LVEF), fractional shortening (FS), left ventricular end-diastolic diameter (LVEDd), and left ventricular end-systolic diameter (LVEDs)-were quantified from M-mode echocardiography. All measurements were performed in triplicate and averaged for analysis.

**Histological and immunofluorescence analysis:** Myocardial fibrosis was assessed by Masson's trichrome staining. Paraffin-embedded cardiac tissue sections were baked overnight at 37 °C, deparaffinized in xylene, and rehydrated through a graded ethanol series. Sections were fixed in Bouin's solution at 56 °C for 90 min, rinsed thoroughly under running tap water, stained with Weigert's hematoxylin for 15 min, and then sequentially stained with acid fuchsin and aniline blue. Differentiation was performed in 1% aqueous acetic acid for 2 min, followed by dehydration through ascending concentrations of ethanol and clearing in xylene. Sections were mounted with neutral balsam. Fibrotic areas were quantified using ImageJ software (National Institutes of Health, USA).

For immunofluorescence staining, tissue sections were washed three times with PBS for 10 min, blocked with 5% normal serum or BSA for 1 h at room temperature. Primary antibodies against α-SMA were applied and incubated overnight at 4 °C. After three 10-min washes with PBS, sections were incubated with fluorophore-conjugated secondary antibodies for 2 h at room temperature in the dark. Nuclei were counterstained with DAPI for 5 min, and coverslips were mounted with antifade mounting medium.

**Evaluation of in vivo therapeutic safety:** Healthy male C57BL/6 mice (6-8 weeks old) were randomly divided into five groups: PBS, CsA, CsA@NPs, CsA@NM-MBs, and CsA@NM-MBs + UTMD. Body weights were recorded on days 0, 7, 14, 21, and 28 to assess systemic toxicity. On day 28 (four weeks after treatment), mice were euthanized by cervical dislocation. Blood were collected via inferior vena cava puncture for hematological and biochemical analyses. Hematological parameters included RBC, white blood cell count (WBC). Biochemical markers included alanine aminotransferase (ALT), aspartate aminotransferase (AST), interleukin-1 beta (IL-1β), tumor necrosis factor alpha (TNF-α), complement component 3a (C3a), complement component 5a (C5a), prothrombin time (PT), activated partial thromboplastin time (APTT). Major organs-including liver, spleen, kidneys, and lungs-were harvested, fixed in 10% neutral-buffered formalin, processed for paraffin embedding, sectioned at 4-5 μm thickness, and stained with hematoxylin and eosin (H&E) for histopathological evaluation.

**Statistical analysis:** Image analysis was performed using ImageJ software (National Institutes of Health, USA). Data were processed and visualized using GraphPad Prism 9.5 (GraphPad Software, San Diego, CA, USA) and OriginPro 2022 (OriginLab Corporation, Northampton, MA, USA). Data conforming to a normal distribution are presented as mean ± standard deviation (mean ± SD). Comparisons between two independent groups were conducted using an unpaired, two-tailed Student's t-test. For comparisons among three or more groups, one-way analysis of variance (ANOVA) was performed, followed by Tukey's multiple comparisons test as a post hoc procedure. Statistical significance was set as *P* < 0.05 (∗*P* < 0.05; ∗∗*P* < 0.01; ∗∗∗*P* < 0.001).

## 3 Results

### 3.1 Preparation and characterization of CsA@NM-MBs motors

**Fig. 1:**
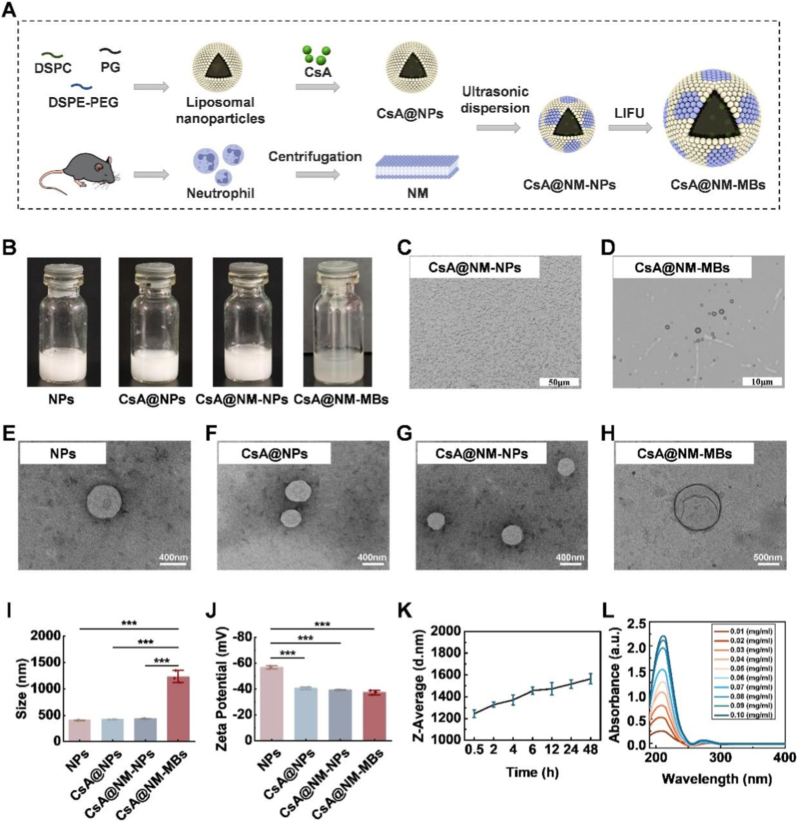
Preparation and characterization of CsA@NM-MBs motors. (A) Schematic illustration of the stepwise preparation procedure for CsA@NM-MBs motors. (B) Morphological characteristics of each group of particles. (C) The light microscopic morphology of CsA@NM-NPs. (D) The light microscopic morphology of CsA@NM-MBs motors. (E-H) Internal structure and morphology of NPs, CsA@NPs, CsA@NM-NPs, CsA@NM-MBs motors observed by TEM. (I) Size of NPs, CsA@NPs, CsA@NM-NPs and CsA@NM-MBs motors. The results are presented as means ± SEM (n = 3, ∗∗∗*p* < 0.001). (J) ζ potential of NPs, CsA@NPs, CsA@NM-NPs and CsA@NM-MBs motors determined by DLS. The results are presented as means ± SEM (n = 3, ∗∗∗*p* < 0.001). (K) The particle size stability of CsA@NM-MBs motors at 48 h. (L) The UV absorption spectra curves of CsA solutions at different concentration gradients.

As illustrated in Fig. 1A, the fabrication of CsA@NM-MBs motors followed a three-step procedure. First, liposomal nanoparticles encapsulating CsA, denoted as CsA@NPs, were synthesized. Next, the CsA@NPs were surface-functionalized via neutrophil membrane camouflage to yield CsA@NM-NPs. Finally, LIFU irradiation was applied to trigger a phase transition, thereby generating CsA@NM-MBs motors. As shown in Fig. 1B, the resulting liposomal dispersions appeared as milky-white suspensions. Light microscopy revealed that CsA@NM-NPs exhibited a spherical morphology with uniform size distribution (Fig. 1C). Following phase transition, CsA@NM-MBs motors displayed an enlarged volume and a characteristic hollow spherical architecture (Fig. 1D). Morphological characterization of NPs, CsA@NPs, CsA@NM-NPs, and CsA@NM-MBs motors was performed using TEM, revealing that all four particle types exhibited uniform size distribution and spherical morphology (Fig. 1E–H). Particle size and zeta potential were analyzed using a Malvern laser particle sizer. The hydrodynamic diameters of NPs, CsA@NPs, CsA@NM-NPs, and CsA@NM-MBs motors were measured as 431.8 ± 12.76 nm, 432.87 ± 2.14 nm, 446.89 ± 2.45 nm, and 1223.34 ± 95.34 nm, respectively (Fig. 1I), while their zeta potentials were determined to be −56.70 ± 0.98 mV, −50.0 ± 0.97 mV, −48.40 ± 1.55 mV, and −47.10 ± 2.35 mV, respectively (Fig. 1J). The particle size of CsA@NM-MBs at different time points within 48 h was measured and its time stability was analyzed. The results showed that CsA@NM-MBs motors were relatively stable within 48 h (Fig. 1K).

The UV absorption spectrum of CsA was determined to identify its maximum absorbance wavelength. A distinct peak was observed at 210 nm (Fig. 1L), which was selected for subsequent quantitative analysis. A standard calibration curve was constructed by plotting CsA concentration (x-axis) against OD210 values (y-axis), yielding equation Y = 27.60X + 0.17 with R^2^ = 0.995, indicating excellent linearity (Fig. S1). Using this curve, EE and LE of CsA@NM-MBs motors were calculated as 67.94 ± 8.41% and 9.11 ± 3.56%, respectively. Following LIFU irradiation, the supernatant was collected and analyzed to quantify the released CsA, revealing a cumulative drug release of 81.35 ± 7.36%.

### 3.2 In vitro assessment of lipid membrane fusion and nanoparticle phase-transition efficiency

**Fig. 2:**
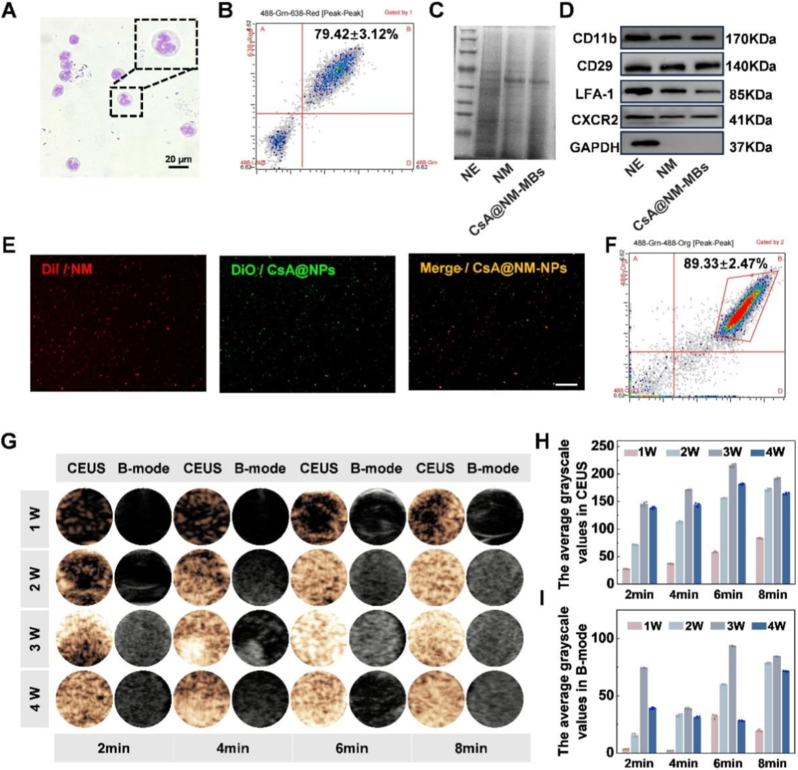
Preparation and characterization of CsA@NM-MBs motors. (A) Optical microscopy images of neutrophils. Inset: Magnified image. (B) Flow cytometric analysis of purity of NE marked by Ly6G Antibody and CD11b Antibody. (C) Protein retention assay of cell membraneon the surface of CsA@NM-MBs motors. (D) The retention of chemotactic and adhesion-related proteins from neutrophil (NE), NM and CsA@NM-MBs motors was detected by WB assay. (E) Co-localization assay of NM and CsA@NPs on the surface of CsA@NM-NPs, scale bar = 50 μm. (F) Fluorescent co-localization of cell membranes and CsA@NPs on the surface of CsA@NM-NPs. (G) Changes in acoustic intensity and analysis in the ultrasound mode of CsA@NM-MBs motors after LIFU irradiation for 2 min, 4 min, 6 min and 8 min. (H) Analysis in the CEUS ultrasound mode of CsA@NM-MBs motors after LIFU irradiation for 2 min, 4 min, 6 min and 8 min. (I) Analysis in the B-mode of CsA@NM-MBs after LIFU irradiation for 2 min, 4 min, 6 min and 8 min.

Wright–Giemsa staining and TEM staining confirmed that the isolated mouse bone marrow-derived neutrophils exhibited the characteristic multilobed nuclear morphology (Fig. 2A and S3). Flow cytometric analysis of neutrophils stained with anti-CD11b and anti-Ly6G antibodies revealed a purity of 79.42 ± 3.12%. (Fig. 2B). To evaluate the preservation of membrane proteins following biomimetic coating, total neutrophil (NE) protein, purified neutrophil membrane (NM) protein, and surface-extracted protein from CsA@NM-MBs motors were subjected to SDS-PAGE followed by Coomassie Brilliant Blue staining. Distinct protein bands corresponding to NM proteins were clearly observed in the CsA@NM-MBs motors lane (Fig. 2C), demonstrating efficient retention of membrane proteins during the coating process. This finding supports both the structural integrity and functional fidelity of the transferred membrane layer. Western blot analysis further confirmed the presence of key functional proteins-including chemotaxis and adhesion-related molecules (CD11b, CXCR2, LFA-1, CD29)-on the surface of CsA@NM-MBs motors (Fig. 2D).

To validate the successful fusion of neutrophil membranes with CsA@NPs, fluorescence co-localization assays were conducted. Specifically, neutrophil membranes were labeled with the lipophilic dye DiI (red fluorescence), while CsA@NPs were labeled with DiO (green fluorescence). Fluorescence imaging analysis revealed extensive overlap between red and green fluorescence signals on CsA@NM-NPs, indicating effective membrane-nanoparticle integration (Fig. 2E). Quantitative analysis yielded a Pearson's correlation coefficient of 0.93, confirming strong linear co-localization (Fig. S2). Flow cytometry further corroborated high fusion efficiency (Fig. 2F).

To evaluate the phase-transition capability of CsA@NM-NPs into microbubbles upon LIFU irradiation, biomimetic nanoparticles were introduced into an in vitro imaging phantom and subjected to LIFU exposed. Acoustic signal enhancement was monitored in both two-dimensional (B-mode) ultrasound and contrast-enhanced ultrasound (CEUS) modes. Ultrasound imaging revealed that CsA@NM-NPs exhibited anechoic or isoechoic characteristics prior to LIFU irradiation. With increasing irradiation duration, a progressive increase in echogenicity was observed in both imaging modalities, with peak signal intensity attained at 6 min (Fig. 2G). Quantitative analysis of mean gray-scale values was performed on representative images acquired under both two-dimensional and CEUS modes using ImageJ software (Fig. 2H and I).

### 3.3 Motion analysis of CsA@NM-MBs motors

**Fig. 3:**
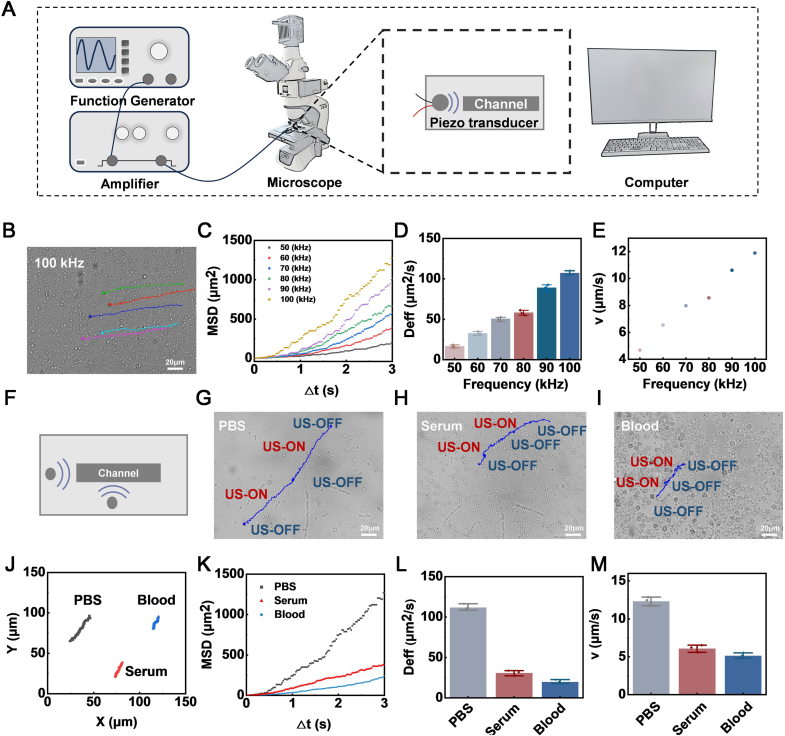
Ultrasonic actuation of the CsA@NM-MBs motors. (A) Schematic illustration of the ultrasonic-driven CsA@NM-MBs motors system. (B) Time-lapse images of the ultrasound-driven CsA@NM-MBs motors. (C) MSD analysis of CsA@NM-MBs motors at different ultrasonic frequency (50, 60, 70, 80, 90 and 100 kHz). (D) Deff of CsA@NM-MBs motors at different ultrasonic frequency (50, 60, 70, 80, 90 and 100 kHz) based on MSD. (E) Velocity of CsA@NM-MBs motors at different ultrasonic frequency (50, 60, 70, 80, 90 and 100 kHz) based on MSD. (F) Schematic illustration of the ultrasonic-driven transducer positioning in different media. (G-I) Time-lapse imaging of the ultrasound-driven CsA@NM-MBs motors in different media under ultrasound irradiation with a 5-s on/5-s off cycling regimen. (J) Representative tracking trajectories of the CsA@NM-MBs motors in different media (PBS, Serum, Blood) within 3 s. (K) MSD analysis of CsA@NM-MBs motor in different media (PBS, Serum, Blood). (L)Deff of CsA@NM-MBs motors in different media (PBS, Serum, Blood) based on MSD (n = 3, ∗∗*p* < 0.01, ∗∗∗*p* < 0.001). (M) Velocity of CsA@NM-MBs motor in different media (PBS, Serum, Blood) based on MSD (n = 3, ∗∗*p* < 0.01, ∗∗∗*p* < 0.001).

Fig. 3A presents a schematic illustration of the ultrasonic driving device. The Trajectory of CsA@NM-MBs motors, recorded over 5 s under 100 kHz ultrasound irradiation, are shown in Fig. 3B. To determine the optimal ultrasonic conditions for propulsion and to evaluate the influence of ultrasound frequency on motility performance, the mean square displacement (MSD) of the CsA@NM-MBs motors was quantified across a frequency range of 50–100 kHz.

A progressive increase in MSD over time intervals was observed at all tested frequencies, with particularly pronounced enhancement at specific frequencies-indicating improved directional movement (Fig. 3C). As shown in Fig. 3D, the effective diffusion coefficient (Deff) of CsA@NM-MBs motors increases from 16.49 ± 1.89 to 107.04 ± 2.59 μm/s^2^ as the ultrasound frequency was raised from 50 to 100 kHz. At 100 kHz, the CsA@NM-MBs motors achieved maximal motility, exhibiting an average velocity of 11.89 μm/s (Fig. 3E).

Fig. 3F presents a schematic illustration of the ultrasonic transducer. Using an ultrasonic field driving device to modulate the on-off cycles of the ultrasound field, ultrasound-mediated control of CsA@NM-MBs motors was monitored under an optical microscope. To comprehensively evaluate the performance of the ultrasonic actuation system, three biologically relevant media—PBS, serum, and whole blood—were selected as propulsion environments. In all three media, the motors exhibited rapid, reproducible activation and initiated stable linear motion immediately upon ultrasound exposure. Upon ultrasound deactivation, motor propulsion ceased instantaneously, and the particles reverted to random Brownian motion (Fig. 3G–I).

To investigate the motility behavior of CsA@NM-MBs motors in different biological media, the motors were separately dispersed in PBS, serum, and blood. Upon ultrasound activation, their motion trajectories, MSD, Deff and velocities were recorded and statistically analyzed over the initial 0–3 s time interval. Representative trajectories of individual CsA@NM-MBs motors in each medium are shown in Fig. 3J. The calculated MSD curves (Fig. 3K) reveal a progressive increase in the slope—indicative of increasingly super-diffusive motion—with increasing medium complexity. Fig. 3L shows that the Deff of CsA@NM-MBs motors in PBS, serum and whole blood are 111.95 ± 4.19, 31.01 ± 3.31 and 20.02 ± 2.41 μm/s^2^, respectively. The movement speed of CsA@NM-MBs motors in PBS is 12.36 ± 2.12 μm/s, in serum is 6.11 ± 2.33 μm/s, and in blood is 5.15 ± 1.19 μm/s. As the medium concentration increases, the movement speed significantly slows down (Fig. 3M).

### 3.4 Assessment of transendothelial barrier penetration

**Fig. 4:**
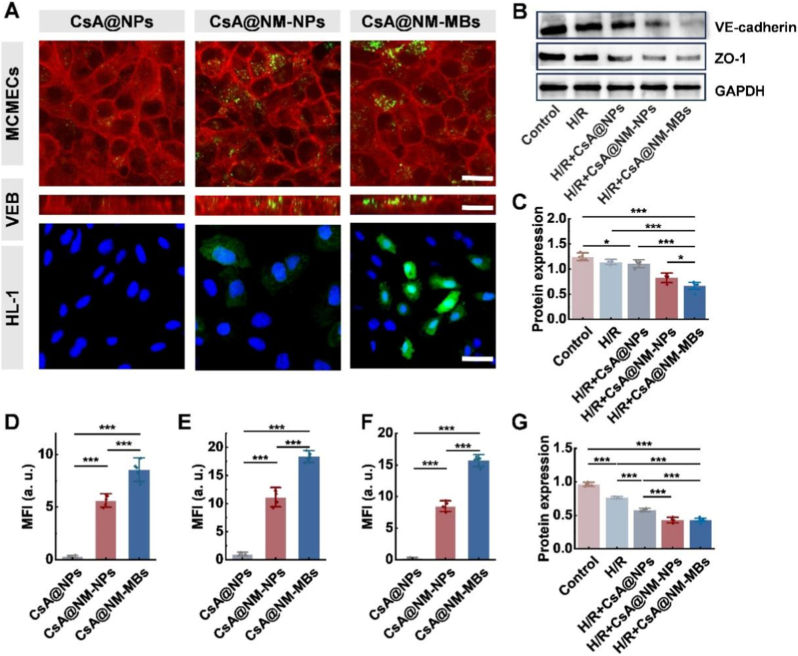
(A). Confocal laser scanning microscope images of the transwell model MCMECs cell layer, vascular endothelial barrier layer, and HL-1 cells. In the images, MCMECs cells were labeled with phalloidin, HL-1 cell nuclei with DAPI, and particles with DiO, scale bar = 10 μm. (B). Expression of VE-cadherin, ZO-1, and GAPDH proteins in endothelial cells after treatment with different particles. (C, G). Statistical results of VE-cadherin and ZO-1 protein expression. (D-F). Fluorescence statistics of the MCMECs cell layer, vascular endothelial barrier layer, and HL-1 cells (n = 5).

To evaluate the capacity of CsA@NM-MBs motors to traverse the vascular endothelial barrier, an in vitro vascular endothelial barrier model was established using a Transwell system. As illustrated in Fig. 4A, MCMECs were seeded in the upper chamber, while HL-1 cardiomyocytes were cultured in the lower chamber. Upon achieving 100% confluence, the MCMEC monolayer was subjected to fMLP added to the lower chamber to mimic an inflammatory microenvironment analogous to that observed in vivo. Subsequently, DiO-labeled CsA@NPs, CsA@NM-NPs, or CsA@NM-MBs were introduced into the upper chamber; in the CsA@NM-MBs motors group, external ultrasound stimulation was applied. The transendothelial penetration efficiency of each formulation was quantitatively assessed by measuring the fluorescence intensity in the lower chamber. As shown in Fig. 4D–F, in the presence of fMLP, neutrophil membrane-coated CsA@NM-NPs exhibited significantly enhanced endothelial barrier penetration compared with controls. Notably, the CsA@NM-MBs motors group—under ultrasound actuation—displayed the strongest fluorescence signal on the HL-1 cell layer in the lower chamber, indicating the highest transendothelial delivery efficiency.

To further investigate treatment-induced alterations in tight junction integrity of the MCMEC monolayer during the Transwell assay, the expression levels of key tight junction proteins were analyzed by Western blotting. As presented in Fig. 4B and C and Fig. 4G, the extent of disruption to the tight junction proteins VE-cadherin and ZO-1 varied markedly across treatment groups. Specifically, the CsA@NM-MBs motors group subjected to ultrasound stimulation induced the most pronounced downregulation and structural disassembly of both VE-cadherin and ZO-1; this was followed by the CsA@NM-NPs group, whereas the CsA@NPs group caused the mildest impairment.

### 3.5 In vitro safety evaluation of CsA@NM-MBs motors

**Fig. 5:**
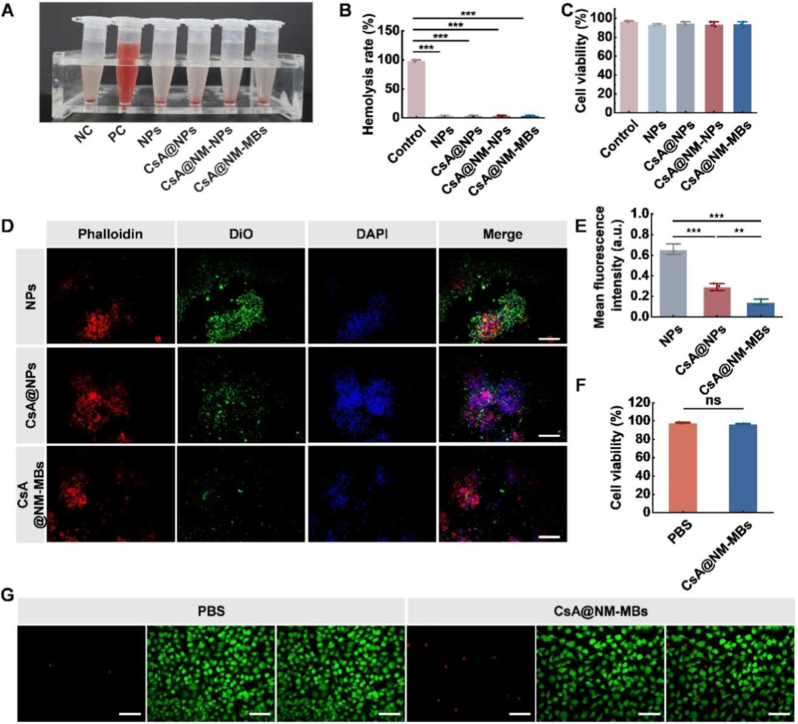
Detection of CsA@NM-MBs motors cytotoxicity and phagocytic escape. (A-B) Analysis of hemolysis rate of NPs、CsA@NPs、CsA@NM-NPs and CsA@NM-MBs motors (n = 3, ∗∗∗*p* < 0.001). (C) Effect of CsA@NM-MBs motors on the viability of H9C2 cells. (D) Fluorescence co-localization assay after co-incubation of NPs, CsA@NPs and CsA@NM-MBs motors with macrophages, scale bar = 20 μm. (E) Fluorescence colocalization analysis in macrophages following co-incubation with NPs, CsA@NPs, and CsA@NM-MBs motors (n = 3, ∗*p* < 0.05). (F) Quantitative analysis of live/dead staining fluorescence in H9C2 cells following PBS and CsA@NM-MBs motors treatment (n = 3, the term “ns” means not significant). (G) Fluorescence images of live/dead staining in H9C2 cells treated with PBS and CsA@NM-MBs motors, scale bar = 20 μm.

To evaluate the in vivo safety of the particles formulations for intravenous administration, their hemocompatibility was assessed. Four formulations-NPs, CsA@NPs, CsA@NM-NPs, and CsA@NM-MBs motors-were tested, yielding hemolysis rates of 3.78 ± 0.91%, 4.08 ± 0.80%, 4.07 ± 0.78%, and 3.22 ± 0.52%, respectively. All values remained below the 5% regulatory threshold for hemolysis, indicating favorable hemocompatibility and supporting the safety of these nanoparticles for intravenous delivery (Fig. 5A and B). Cell viability was evaluated using the CCK-8 assay to determine the impact of each particle formulation on H9C2 cells. Cell viability exceeded 90% across all treatment groups, demonstrating excellent in vitro biocompatibility of the particles system (Fig. 5C).

To investigate the capacity of neutrophil membrane-coated CsA@NM-MBs motors to evade macrophage phagocytosis, NPs, CsA@NPs, and CsA@NM-MBs motors were fluorescently labeled with DiO (green). These particles were co-incubated with macrophages for 2 h, followed by nuclear staining with DAPI. Fluorescence microscopy was employed to visualize particle adhesion and internalization, thereby enabling a comparative assessment of macrophage uptake efficiency across the different formulations (Fig. 5D and E). Significantly lower fluorescence intensity was observed in macrophages treated with CsA@NM-MBs relative to those exposed to either bare NPs or CsA@NPs, confirming that neutrophil membrane coating enhances immune evasion by attenuating recognition and subsequent phagocytosis by the mononuclear phagocyte system.

To further evaluate the biocompatibility of CsA@NM-MBs motors, H9C2 cells were co-incubated with either PBS or CsA@NM-MBs motors and subjected to live/dead cell staining. Quantitative analysis of the viable-to-dead cell ratio revealed survival rates exceeding 95% in both groups, demonstrating excellent cellular safety and negligible cytotoxicity of the motors formulation (Fig. 5F and G).

### 3.6 In vitro functional assessment of CsA@NM-MBs motors

**Fig. 6:**
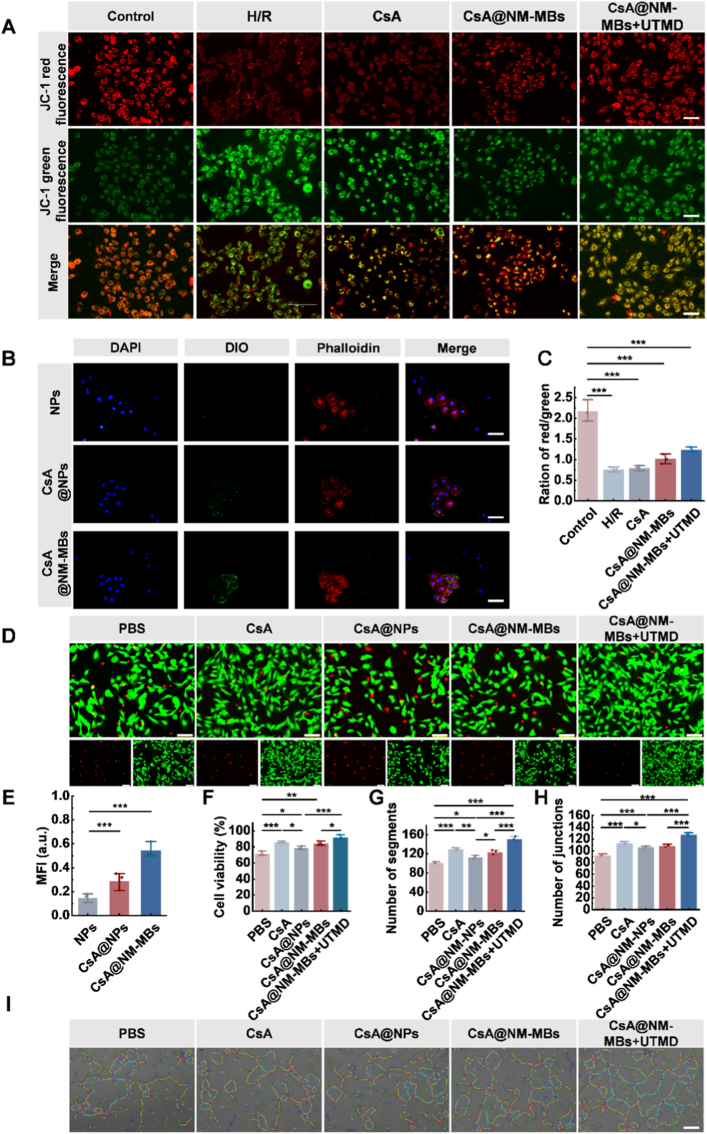
(A) JC-1 fluorescence staining for mitochondrial membrane potential in H9C2 cells with H/R injury following treatment with CsA, CsA@NM-MBs, or CsA@NM-MBs + UTMD, scale bar = 20 μm. (B) Fluorescence colocalization analysis following co-incubation of NPs, CsA@NPs, or CsA@NM-MBs with inflamed endothelial cells, scale bar = 20 μm. (C) Quantitative analysis of JC-1 fluorescence staining for mitochondrial membrane potential recovery in H9C2 cells following H/R injury and treatment with CsA, CsA@NM-MBs, or CsA@NM-MBs + UTMD (n = 3, ∗*p* < 0.05, ∗∗∗*p* < 0.001). (D) Representative images of live/dead staining in H9C2 cells following H/R injury and co-culture with CsA, CsA@NPs, CsA@NM-NPs, or CsA@NM-MBs + UTMD treatment scale bar = 20 μm. (E) Quantitative analysis of fluorescence colocalization analysis following co-incubation of NPs, CsA@NPs, or CsA@NM-MBs with inflamed endothelial cells (n = 3, ∗∗∗*p* < 0.001). (F) Quantitative analysis of live/dead staining in H9C2 cells following H/R injury and co-culture with CsA, CsA@NPs, CsA@NM-MBs, or CsA@NM-MBs + UTMD treatment (n = 3, ∗∗*p* < 0.01, ∗∗∗*p* < 0.001). (G). Quantitative analysis of the number of segments in endothelial cell-mediated angiogenesis was performed for each experimental group (n = 3, ∗*p* < 0.05, ∗∗*p* < 0.01, ∗∗∗*p* < 0.001). (H). Quantitative analysis of the number of junctions in endothelial cell-mediated angiogenesis was performed for each experimental group (n = 3, ∗*p* < 0.05, ∗∗∗*p* < 0.001). (I) Tube formation capacity of endothelial cells was evaluated by optical microscopy following treatment with CsA, CsA@NPs, CsA@NM-MBs, or CsA@NM-MBs + UTMD, scale bar = 20 μm.

The JC-1 assay was employed to evaluate the impact of CsA@NM-MBs motors combined with UTMD on mitochondrial membrane potential in H9C2 cardiomyocytes subjected to hypoxia-reoxygenation (H/R) injury. A significantly decreased red-to-green fluorescence intensity ratio was observed in the H/R injury group, indicating mitochondrial membrane depolarization under ischemic stress. Pretreatment with CsA, CsA@NM-MBs, or CsA@NM-MBs + UTMD all increased the red/green fluorescence ratio; however, the most substantial restoration was achieved in the CsA@NM-MBs + UTMD group. These findings suggest that ultrasound-targeted delivery potentiates the protective effect of CsA on mitochondrial function in H/R-injured cardiomyocytes (Fig. 6A–C).

To assess the endothelial targeting capability of the particles, DiO-labeled NPs, CsA@NPs, and CsA@NM-MBs were co-incubated with inflammatory endothelial cells. Cell nuclei were counterstained with DAPI and F-actin was labeled with phalloidin for fluorescence microscopy analysis. A markedly higher number of CsA@NM-MBs adhered specifically to the inflamed endothelial surfaces-attributable to neutrophil membrane-derived integrins displayed on their surface, which recognize upregulated adhesion molecules on activated endothelium. In contrast, NPs and CsA@NPs-lacking this biomimetic ligand display-exhibited significantly lower binding affinity. These results confirm that CsA@NM-MBs possess enhanced and selective adhesion to inflammatory endothelial cells (Fig. 6B and E).

In the context of myocardial ischemia-reperfusion injury, hypoxia-reoxygenation induces severe cellular damage and functional impairment, resulting in markedly reduced cell viability and an elevated risk of apoptosis or necrosis. To evaluate the cytoprotective efficacy of various therapeutic formulations, we assessed the effects of CsA, CsA@NPs, CsA@NM-MBs, and CsA@NM-MBs + UTMD on H/R-injured cardiomyocytes. Relative to the untreated control group, treatment with CsA or CsA@NPs conferred moderate improvement in cell viability and a reduction in dead cells (red fluorescence). Notably, the CsA@NM-MBs group exhibited the most pronounced cytoprotective effect, characterized by the highest proportion of viable cells (green fluorescence) and minimal cell death-thereby demonstrating superior cardioprotection (Fig. 6D and F).

We further assessed the influence of CsA, CsA@NPs, CsA@NM-MBs, and CsA@NM-MBs + UTMD on the angiogenic tube formation capacity of myocardial cells following H/R injury. In the control group, tube formation was sparse, short, and structurally incomplete. The CsA group showed modest improvement in tubular network development. Tube formation was further enhanced in the CsA@NPs group, with increased number, length, and structural regularity of capillary-like structures. Most strikingly, the CsA@NM-MBs + UTMD group displayed the most robust angiogenic response, forming extensive, interconnected, and morphologically mature tubular networks (Fig. 6I), indicating superior pro-angiogenic activity(Fig. 6G–I).

### 3.7 In vivo evaluation in murine MIRI models

**Fig. 7:**
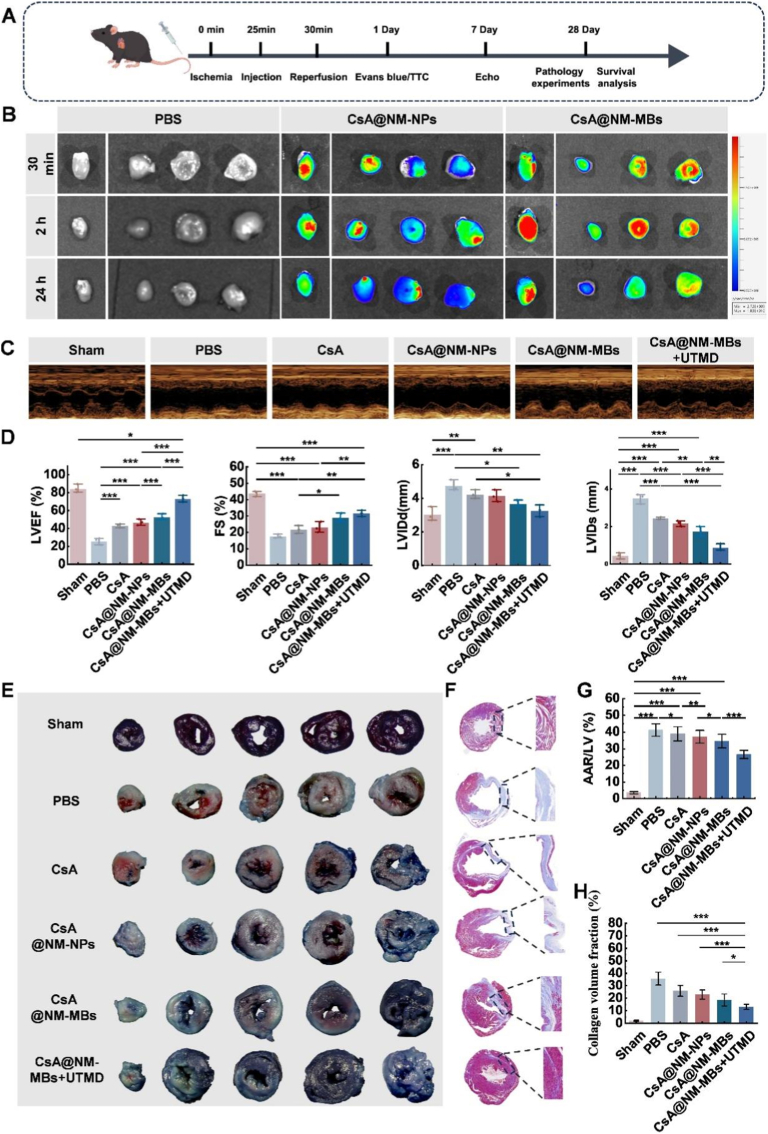
(A) Evaluation of in vivo therapeutic effect of CsA@NM-MBs + UTMD. (B) Distribution detection of particles in myocardium at different time points. (C) Schematic of representative M-mode ultrasound cardiac function test 7 days after MIRI. (D) Quantitative analysis of LEVF (%), FS (%), LVIDd (mm), LVIDs (mm) (n = 3, ∗*p* < 0.05, ∗∗*p* < 0.01 ∗∗∗*p* < 0.001). (E) Evans blue and TTC double staining in each treatment group. (F) Representative image of Masson staining 28 days after MIRI. (G) Quantitative analysis of collagen fiber content within the myocardium (n = 3, ∗*p* < 0.05, ∗∗∗*p* < 0.001). (H) Quantitative analysis of Evans blue and TTC double staining (n = 3, ∗*p* < 0.05, ∗∗∗*p* < 0.001). (For interpretation of the references to color in this figure legend, the reader is referred to the Web version of this article.)

To further validate the therapeutic effect of CsA@NM-MBs in murine MIRI models, the experimental timeline is illustrated in Fig. 7A. Cardiac biodistribution of CsA@NM-NPs and CsA@NM-MBs was evaluated using a small-animal in vivo imaging system. Following intravenous administration via the tail vein, both formulations rapidly accumulation in the heart within 30 min, with peak fluorescence signal intensity observed at 2 h post-injection. By 24 h, cardiac fluorescence signals had markedly declined, indicating progressive systemic clearance. Notably, ultrasound-driven CsA@NM-MBs demonstrated significantly enhanced cardiac accumulation compared with CsA@NM-NPs group, (Fig. 7B, Fig. S4). To assess whether the pharmacokinetic profile and tissue distribution of CsA were consistent with those of its fluorescently labeled analogs, equivalent doses of free CsA, CsA@NM-NPs, and CsA@NM-MBs were administered intravenously to mice subjected to MIRI. Blood and major organ samples were collected at predetermined time points post-reperfusion. Pharmacokinetic analysis revealed that CsA@NM-MBs conferred significantly higher and more sustained CsA accumulation in both blood and cardiac tissue of MIRI mice compared with the other formulations (Fig. S5–S6). The distribution of CsA@NM-MBs in the liver, kidneys and spleen was less than that of the CsA@NM-NPs group (Fig. S7). Inflammation-, chemotaxis-, and ultrasound-mediated targeting significantly enhanced the cardiac biodistribution of CsA@NM-MBs in mice with myocardial ischemia–reperfusion injury, enabling selective accumulation at the sites of myocardial damage.

Cardiac systolic function was assessed by echocardiography at 7 days post-myocardial infarction (Fig. 7C) to evaluate the therapeutic efficacy of different treatment regimens. The sham-operated group exhibited normal LVEF of 87.25 ± 1.59%, whereas the PBS-treated group displayed severe systolic dysfunction, with LVEF reduced to 42.54 ± 2.81%, accompanied by impaired left ventricular wall motion and chamber dilation. Among all groups, the CsA@NM-MBs + UTMD group displayed the most robust functional recovery, achieving an LVEF of 73.32 ± 3.74% and FS of 41.82 ± 2.14%. Comprehensive echocardiographic analysis confirms that CsA@NM-MBs + UTMD effectively ameliorates cardiac dysfunction following MIRI (Fig. 7D).

Prior to reperfusion in murine models of MIRI, particles from each experimental group were administered via tail vein injection. On day 1 post-treatment, hearts were harvested and subjected to dual staining with Evans blue and TTC to delineate the area at risk (AAR) (Fig. 7E). Quantitative analysis of the left ventricular AAR revealed that the CsA@NM-MBs + UTMD treatment group exhibited a significantly reduced AAR proportion compared to both the CsA@NM-MBs and CsA@NM-NPs groups (Fig. 7G). Collectively, these results demonstrate that ultrasound-triggered CsA@NM-MBs + UTMD motors therapy effectively reduces myocardial infarction area in MIRI mice.To evaluate the therapeutic impact of CsA@NM-MBs motors on myocardial fibrosis in murine models of MIRI, Masson's trichrome staining was performed to assess collagen deposition and fibrotic area in cardiac tissues four weeks after acute myocardial infarction (Fig. 7F). The results revealed that, with the exception of the sham-operated group, all experimental groups exhibited blue-stained collagen fiber accumulation within the infarcted regions. The PBS-treated group displayed the most extensive fibrosis accompanied by substantial loss of cardiomyocytes. In contrast, the CsA@NM-MBs group showed no significant reduction in fibrotic area, whereas the CsA@NM-MBs + UTMD treatment group demonstrated a marked decrease in myocardial fibrosis compared to the other four groups (Fig. 7H).

**Fig. 8:**
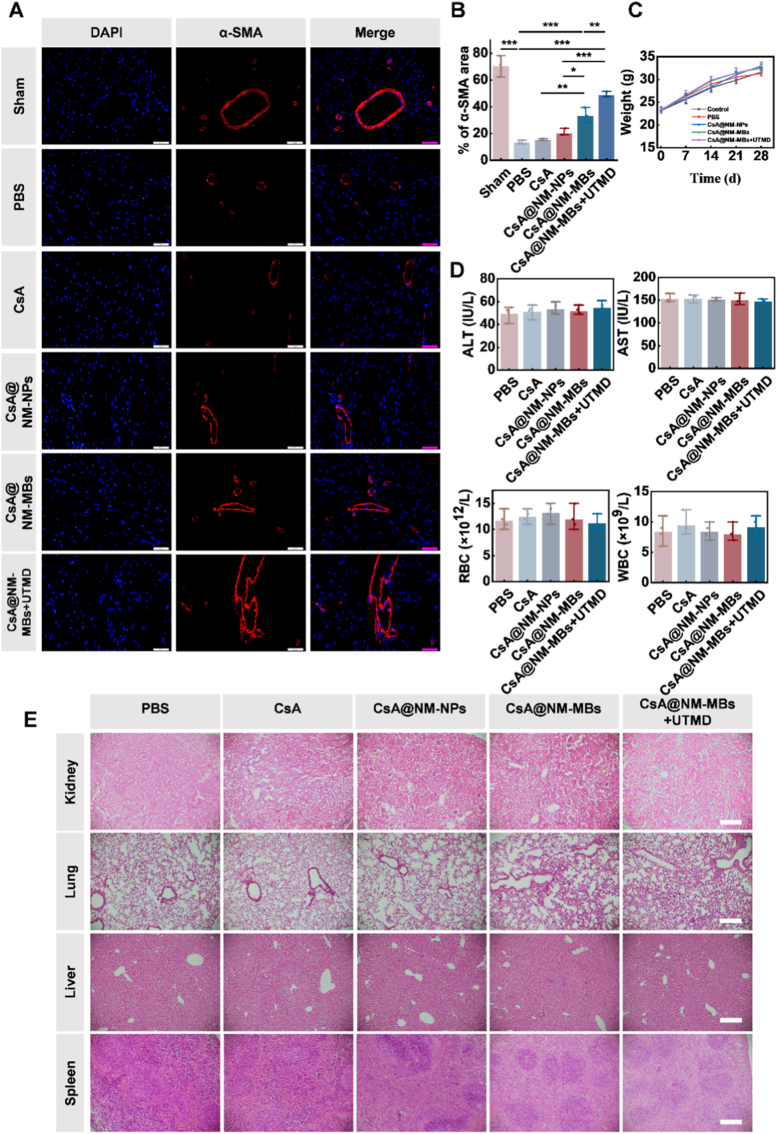
(A) Immunofluorescence staining was performed on frozen sections of mouse hearts 28 days after MIRI to detect microvascular density in the ischemic border zone, scale bar = 50 μm; (B) Semi-quantitative analysis of α-SMA cell fluorescence intensity (n = 4, ∗*P* < 0.05, ∗∗*P* < 0.01, ∗∗∗*P* < 0.001). (C) Body weight change curves for mice in each group (n = 3). (D) Analysis of blood routine and main biochemical indicators in the liver and kidneys (n = 3). (E) Observation of lung, liver, spleen, and kidney structures using H&E staining in mice from each group. No significant tissue damage like hemorrhage, edema, or inflammatory infiltration was observed, scale bar = 50 μm.

Immunofluorescence staining was performed on frozen heart sections from mice after MIRI to evaluate α-SMA expression across treatment groups(Fig. 8A). The CsA@NM-MBs + UTMD group exhibited the highest microvascular density compared to the other groups (Fig. 8B), indicating enhanced angiogenic activity and supporting the conclusion that this treatment promotes the maturation of newly formed blood vessels.

Body weight was monitored over 28 days post-treatment, and all groups of mice exhibited stable and continuous weight gain (Fig. 8C). In this study, the peripheral blood indicators measured were divided into four categories: routine biochemical parameters, inflammatory and immune molecular indicators, coagulation function indicators, and blood cell parameters. There were no significant differences among the various parameters (Fig. 8D–S8).

Four weeks after MIRI induction, major organs—including the heart, liver, spleen, lungs, and kidneys—were harvested and subjected to H&E staining to assess potential systemic toxicity. Histopathological examination revealed no notable abnormalities in any of the examined organs. Specifically, there was no evidence of hemorrhage, edema, or inflammatory cell infiltration, demonstrating that the formulated particles do not induce detectable organ damage at the therapeutic dose used in this study and exhibit a favorable safety profile (Fig. 8E).

## 4 Discussion

In this study, we developed an ultrasound-powered biomimetic microbubble motor system (CsA@NM-MBs motors) that synergistically combines the innate chemotactic capabilities of neutrophil membranes with the ultrasound-triggered directional propulsion of lipid-based microbubbles to enable active, targeted therapy for MIRI. During delivery of the immunosuppressant CsA, this system sequentially overcomes critical physiological barriers-including immune clearance in systemic circulation, physical obstruction by the vascular endothelial barrier, and the inherently low permeability of cardiomyocyte membranes—thereby demonstrating excellent biocompatibility, spatiotemporally controllable motility, and robust therapeutic efficacy.

First, the neutrophil membrane confers intrinsic chemotactic functionality, enabling the motor to actively home to inflammatory sites [24]. By inheriting the natural ability of neutrophils to sense gradients of pro-inflammatory cytokines [25], the CsA@NM-MBs motors autonomously migrate toward the injured myocardium. In this work, liposomal cores were coated with neutrophil membranes via ultrasonic vibration—a method that successfully preserved key membrane proteins on the liposome surface [26]. Western blot analysis confirmed the retention of functional receptors, including CXCR2 and CD11b. Consistent with prior reports on cell membrane–coated gold nanorobots, our system exhibited markedly reduced phagocytic uptake in macrophage evasion assays and maintained colloidal stability—with negligible changes in particle size over 48 h. This design effectively circumvents a fundamental limitation of conventional liposomes: rapid opsonization and clearance by the mononuclear phagocyte system—thus establishing a critical foundation for prolonged systemic navigation and precise, site-specific accumulation in vivo [27,28].

Second, the ultrasound-driven mechanism enables controllable, directional motion in physiological environments. We systematically evaluated the motor's responsiveness to ultrasound stimulation, its propulsion velocity, and its motion controllability. The CsA@NM-MBs motors exhibited robust ultrasound responsiveness and precise directional control across multiple physiological media—including PBS, serum, and whole blood. Upon activation of the ultrasound field, the motors initiated unidirectional forward propulsion; upon deactivation, propulsion ceased immediately, and the particles reverted to random Brownian motion. This behavior of ultrasound-driven microbubble movement is consistent with the research results of Fonseca et al.—affords precise temporal control over motor “start–stop” dynamics, thereby offering a reliable strategy for regulating motion [14,29] during targeted delivery. At the optimal excitation frequency of 100 kHz, the maximum propulsion speed reached 12.36 μm/s in PBS; speeds of 6.11 μm/s and 5.15 μm/s were sustained in serum and whole blood, respectively. It should be noted that this study has certain limitations. Specifically, the motility performance of the CsA@NM-MBs motors was assessed primarily under in vitro static or low-resistance chamber conditions; their in vivo motility behavior and targeting efficiency—particularly under physiologically relevant hemodynamic conditions involving blood flow–induced shear stress and erythrocyte crowding—have not yet been systematically validated. Future work should integrate in vivo imaging modalities with biomechanical modeling to further elucidate the navigation capability and spatiotemporally resolved drug release kinetics of these micromotors within dynamic vascular networks.

Transwell assays further confirmed the superior capability of CsA@NM-MBs motors to penetrate the endothelial barrier. Compared with the CsA@NPs group, the motors demonstrated significantly enhanced transendothelial migration efficiency under ultrasound activation. In vivo cardiac imaging further revealed that ultrasound-driven propulsion markedly increased motor accumulation in ischemic myocardial regions. Specifically, in the ultrasound-treated group, fluorescence intensity in the cardiac region was approximately 1.37-fold higher than that observed in the non-ultrasound group. In contrast, the non-ultrasound group relied predominantly on passive, slow chemotactic migration, resulting in limited accumulation at the target site. These findings collectively confirm that ultrasound-driven directional propulsion substantially enhances the targeted accumulation of motors in diseased organs. Echocardiography and Evans Blue/TTC dual staining further substantiated the therapeutic efficacy of the motors against MIRI. Relative to the model control group, the ultrasound-driven group exhibited significant improvements in key cardiac functional parameters—including LVEF and FS—along with a pronounced reduction in the infarct size ratio (i.e., the ratio of the TTC-stained pale area to the Evans Blue–unstained area-at-risk). Notably, the combination of ultrasound-driven propulsion and UTMD yielded the most robust therapeutic outcome among all treatment groups. Although these results provide compelling evidence for the therapeutic potential of CsA@NM-MBs motors, it is important to acknowledge that most in vivo experiments were conducted with a small sample size of only three animals per group (n = 3). Such limited replication may constrain statistical power and reduce the precision of effect size estimates. Therefore, future studies employing larger, adequately powered cohorts are warranted to rigorously validate the robustness and generalizability of these findings.

In conclusion, this study successfully developed a biomimetic drug delivery system that actively traverses multiple physiological barriers by synergistically integrating the innate chemotactic properties of neutrophil membranes with ultrasound-responsive microbubble-based micromotors. The platform enabled efficient, targeted delivery of CsA in a murine model of MIRI, resulting in significant improvement in cardiac function. Moreover, it represents a programmable and modular nanotechnological strategy with broad translational potential for precision therapy of diverse pathological conditions.

## CRediT authorship contribution statement

**Mingyuan Xu:** Conceptualization, Data curation, Formal analysis, Methodology, Software, Visualization, Writing – original draft, Writing – review & editing. **Jiaxin Shan:** Writing – review & editing. **Haichao Yang:** Methodology. **Jiaxu Wang:** Resources. **Haobo Yang:** Data curation. **Jinhong Liu:** Investigation, Methodology. **Jianfeng Chen:** Methodology. **Dandan Yu:** Formal analysis. **Tiecheng Zhang:** Methodology. **Meng Yang:** Investigation. **Wei Zhang:** Software. **Zhuo Wang:** Funding acquisition. **Xiaoping Leng:** Conceptualization, Funding acquisition, Methodology.

## Declaration of competing interest

The authors declare that they have no known competing financial interests or personal relationships that could have appeared to influence the work reported in this paper.

## Data Availability

Data will be made available on request.
