# Supplementary material for: Ultrasound-driven microbubble motors for targeted myocardial ischemia-reperfusion injury treatment
Source: Mater Today Bio. 2026 Jun 13;39:103349. doi: 10.1016/j.mtbio.2026.103349 (PMC13293769; doi:10.1016/j.mtbio.2026.103349)
Supplement: Multimedia component 1 [file mmc1.docx]

**Supplementary Information**

**Ultrasound-Driven Microbubble Motors for Targeted Myocardial Ischemia-Reperfusion Injury Treatment**

**
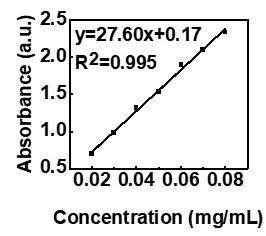
**

**Fig. S1. The CBD standard curve.**

**
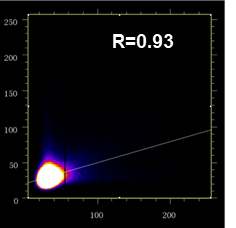
**

**Fig. S2. The Pearson correlation coefficient of fluorescence co-localization of NM and CsA@NPs.**

**Fig. S3. Confocal microscope images of neutrophils.**

**Fig. S4. Quantitative analysis of particles in myocardium at different time points.**

| **Pharmacokinetic parameters** | **CsA** | **CsA@NM-NPs** | **CsA@NM-MBs** |
| --- | --- | --- | --- |
| **AUC(0-t), ng/ml·h** | 1208.57 ± 108.66 | 2677.07 ± 200.46 | 3847.70 ± 103.04 |
| **AUC(0-∞), ng/ml·h** | 1347.22 ± 109.73 | 3640.60 ± 330. 68 | 4997.83 ± 328.73 |
| **t_1/2_, h** | 5.21 ± 1.15 | 6.87 ± 2.05 | 7.87 ± 2.63 |
| **CL, L/h/kg** | 1865.37 ± 148.90 | 691.53 ± 66.54 | 501.91 ± 32.20 |
| **Vd, L/kg** | 12.35 ± 0.48 | 7.84 ± 0.37 | 5.43 ± 0.14 |

**Fig. S5. Quantification of the concentration of CsA in blood after injection of free CsA, CsA@NM-NPs, or CsA@NM-MBs into MIRI mice (n = 5).**

**Fig. S6. Quantification of the concentration of CsA in blood after injection of free CsA, CsA@NM-NPs, or CsA@NM-MBs into MIRI mice (n = 5).**

**Heart**

**Liver**

**Kidney**

**Spleen**

**Fig. S7. Quantification of the concentration of CsA in heart, liver, kidney, and spleen after injection of free CsA, CsA@NM-NPs, or CsA@NM-MBs into MIRI mice (n = 5).**

**Fig. S8. Analysis of blood routine and main biochemical indicators.**
